# From Guided Surgery to Final Prosthesis with a Fully Digital Procedure: A Prospective Clinical Study on 15 Partially Edentulous Patients

**DOI:** 10.1155/2016/7358423

**Published:** 2016-07-14

**Authors:** Giorgio Andrea Dolcini, Marco Colombo, Carlo Mangano

**Affiliations:** ^1^Private Practice, 21100 Como, Italy; ^2^Private Practice, 21052 Busto Arsizio, Italy; ^3^Department of Dental Sciences, University Vita Salute San Raffaele, 20132 Milan, Italy

## Abstract

*Scope.* To demonstrate guided implant placement and the application of fixed, implant-supported prosthetic restorations with a fully digital workflow.* Methods.* Over a 2-year period, all patients with partial edentulism of the posterior maxilla, in need of fixed implant-supported prostheses, were considered for inclusion in this study. The protocol required intraoral scanning and cone beam computed tomography (CBCT), the superimposition of dental-gingival information on bone anatomy, surgical planning, 3D-printed teeth-supported surgical templates, and modelling and milling of polymethylmethacrylate (PMMA) temporaries for immediate loading. After 3 months, final optical impression was taken and milled zirconia frameworks and 3D-printed models were fabricated. The frameworks were veneered with ceramic and delivered to the patients.* Results.* Fifteen patients were selected for this study. The surgical templates were stable. Thirty implants were placed (BTK Safe®, BTK, Vicenza, Italy) and immediately loaded with PMMA temporaries. After 3 months, the temporaries were replaced by the final restorations in zirconia-ceramic, fabricated with a fully digital process. At 6 months, none of the patients reported any biological or functional problems with the implant-supported prostheses.* Conclusions.* The present procedure for fully digital planning of implants and short-span fixed implant-supported restorations has been shown to be reliable. Further studies are needed to validate these results.

## 1. Introduction

Digital workflow has an increasingly important role to play in contemporary dentistry [1, 2].

The advantage of guided implant surgery is that the implant is placed in a safer, more predictable manner, using a surgical template designed and produced using computer-aided-design/computer-aided-manufacturing (CAD/CAM) technology; this prosthetically guided placement is achieved using software for virtual implant planning [2, 3]. Guided implant surgery can also help the dentist to perform flapless implant surgery with less discomfort for the patient and faster working and healing times [2, 3].

Digital scanning and Cone Beam Computer Tomography (CBCT) are the procedures now used for digital workflow for planning guided implant surgery [4, 5]. Taking optical impressions with powerful intraoral scanners for fabricating permanent prostheses on natural teeth and dental implants is becoming widespread and has many advantages over the conventional way of taking impressions, involving less discomfort for the patient, as well as greater speed, accuracy, precision, and reproducibility [6–9]. Optical impression-taking enables collection of all the three-dimensional (3D) information of dentogingival tissues [7, 8]. CBCT on the other hand allows collection all 3D information on the anatomy of the residual crest bone, including height, thickness, and angle [4, 10].

The composition and superimposition of dental and gingival information acquired by intraoral scanning, as well as bone information acquired by CBCT, now allow virtual planning for placing the implants, fabricating the templates for guided surgery, and modelling and preparing temporaries for immediate loading [11, 12].

The purpose of this study is to demonstrate guided implant placement and the application of fixed, implant-supported prosthetic restorations carried out with fully digital workflow. For this purpose, intraoral scanning techniques, virtual planning, computer guided surgery, and immediate loading protocol for the temporary prostheses have been used.

## 2. Materials and Methods

### 2.1. Selection of Patients

In the period between January 2014 and January 2015, all patients seen at two private dental clinics who presented with partially edentulous posterior maxilla and requested restoration of masticatory efficiency with an implant-supported fixed prosthesis were considered for inclusion in this study. The criteria for inclusion consisted of (1) partially edentulous posterior areas (premolars/molars) of the maxilla, (2) sufficient bone for the placing of implants at least 3.75 mm in diameter and 8.0 mm in length, and (3) willingness to participate fully in the protocol. Excluded from the study were (1) patients with systemic diseases having contraindications to implant surgery (e.g., uncontrolled diabetes, blood diseases, and psychiatric illnesses), (2) patients undergoing chemotherapy and/or radiotherapy, (3) patients receiving immunosuppressive therapies, (4) patients being treated with bisphosphonates orally and/or parenterally, (5) patients with active oral or periodontal infections (pus, fistulas, and periodontal abscesses), (6) patients with other oral diseases (vesiculobullous and ulcerative diseases, red and white lesions, and diseases of the salivary glands and cystic lesions), (7) patients with a poor oral hygiene, (8) patients with restricted mouth openings, functional limitations, or temporomandibular disorders, (9) smokers, and (10) bruxists. The protocol for this study was explained in detail to each patient, who signed an informed consent to the implant treatment. The study was carried out in accordance with the protocols established by the 1975 Helsinki Declaration (2008 review).

### 2.2. Image Acquisition

A full examination of soft and hard tissue was performed on each patient. Specifically, in a single appointment designated exclusively to image acquisition, each patient underwent optical scanning with a powerful intraoral scanner (Trios®, 3-Shape, Copenhagen, Denmark) and X-ray examination with CBCT (CS 9300®, Carestream Health, Rochester, NY, USA). In detail, the first examination that patients underwent was an intraoral scan of both arches, including scanning of the bite. This scan was performed after placing a number of moderately radiopaque markers (at least 3) on the teeth adjacent to the edentulous section, using a modified glass ionomer cement (Ketac Cem Radiopaque®, 3M ESPE, St Paul, MN, USA). Particular care was taken when scanning the teeth adjacent to the edentulous section and surrounding soft tissue. The patient underwent a CBCT scan immediately after, with the radiopaque markers still in place. A field of view (FOV) of 10 × 5 cm was adopted, to enable a sufficient amount of data to be collected that could also be superimposed. At this point, the owners' files generated by the intraoral scan and the Digital Imaging and Communication in Medicine (DICOM) files generated by the CBCT were transformed into solid-to-layer files (STL) and sent to a service centre (BTK Guided Surgery®, BTK, Dueville, Vicenza, Italy), for the case to be planned. The patient was then discharged after removal of the radiopaque markers.

### 2.3. Image Processing and the Guided Surgery Project

The STL files obtained from intraoral scanning were superimposed on the STL files obtained from the reconstruction of CBCT with proprietary software (BTK Guided Implant Planning®, BTK, Dueville, Vicenza, Italy) for guided surgery planning. Superimposition was obtained as follows. First, the intraoral scan model was roughly superimposed to the CBCT model using a “three-point” registration tool. After this first rough alignment, the final registration was performed using a “best fit alignment” function. The resulting superimposed models were then used to design teeth-supported surgical templates. The placing of the implants was thus planned virtually, taking into account the position, depth, and angle inside the residual bone crest, and we then proceeded to model the immediate temporary prostheses to be placed* in situ* on the day of the surgery. The planning was sent to the dentist for approval and implementation of any modifications required. Once the plan was confirmed and checked, the service centre physically fabricated the templates for the guided surgery in transparent acrylic resin using 3D printing; the temporary prostheses were milled from polymethylmethacrylate (PMMA). The prostheses were delivered to the dentist together with the guided implant surgery kit, the provisional titanium abutments, and the surgical guides or templates for implant placement.

### 2.4. Surgery and Immediate Loading

Before the operation the patient's mouth was rinsed with a solution of chlorhexidine digluconate 0.2% for 2 minutes. A local anesthetic was obtained with mepivacaine (4% infiltration with epinephrine 1 : 100,000). The teeth-supported surgical template was positioned and the intervention was ready to begin. The surgery was performed with a minimally invasive flapless procedure, without lifting the mucoperiosteal flap. The first step was to remove tissue overgrowth with a punch to allow access to the underlying bony crest, proceeding to preparation of the implant sites using drills of increasing diameters, guided in terms of placement, angle, and depth by the surgical guide. In particular, control of the angle and depth was achieved with a series of diameter reducers positioned inside the drill bushing. In effect, as the size of the drill increased, the diameter reducers were changed until the final diameter was reached, as determined in the surgery planning. Only internal hex implants were used (BTK Safe, BTK, Dueville, Vicenza, Italy), with a diameter of 3.75 mm and length of 8.0 mm, 10.0 mm, or 12.0 mm. Insertion of the implant, clamping, and tightening were performed with a torque wrench through the template, hence with the surgical guide in position. On completion of implant placement the guide was removed from the oral cavity. The dentist was able to check the depth of the implants in relation to the mucosa. X-rays of the intraoral implants were taken straight away and the temporary titanium abutments and temporary PMMA restorations were adjusted. The restorations were positioned without the need for relining. Once any slight frictions were removed, the restorations were polished and screwed onto the abutments. The occlusal access hole was provisionally closed with composite resin. The occlusion was carefully checked using occlusal registration paper. The patient was then discharged with a prescription for antibiotics (amoxicillin clavulanic acid, 1 g every 12 hours for a total of 6 days) and analgesic (ibuprofen 600 mg for a total of three days). The patient was asked to rinse with chlorhexidine gluconate 0.2% 2-3 times a day for 4-5 days following the surgery and to avoid chewing hard foods for a period of 1 week.

### 2.5. Final Prosthesis

The PMMA temporaries were left in place for a total period of 3 months; after this, the patients were recalled for a second round of intraoral imaging, required for modelling and fabrication of the final prostheses. This scan was performed after removing the temporary restorations and abutments and subsequent positioning of scanbodies in polyether-ether-ketone (PEEK). These transfer devices were employed for their ideal optical characteristics, since they do not reflect light as metals do and therefore allow the exact position of the implants to be detected. The abutments and temporary prostheses were then replaced and the patient was discharged. The final scan was then converted into an STL file which was sent to the laboratory and used to determine the exact spatial location of the implants and planning of the final prosthetic structure (zirconia framework). The framework was milled in zirconia, tried in a model of the patient's mouth created with a 3D printer, and sent to the clinician for trying in the oral cavity. The model was 3D-printed in resin. During the trial, the dentist was able to assess the quality of marginal fit of the zirconia framework on the final abutments. At this point the framework was sent back to the laboratory for finalisation, that is, veneering with ceramic. The patient was called in one week later to have the final, zirconia-ceramic restoration delivered. Occlusion was checked using registration papers, and the patient was then discharged with his/her final restoration, which was cemented onto the screw-in abutments with a small amount of zinc oxide eugenol cement.

## 3. Results

**Figure 1:**
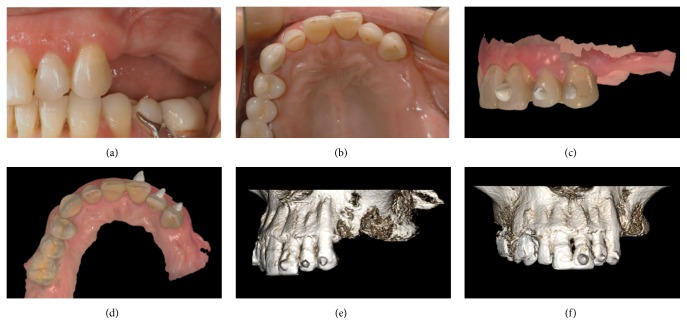
Image acquisition: (a) preoperative clinical picture, side view; (b) preoperative clinical picture, occlusal view; (c) intraoral scan with references, side view; (d) intraoral scan with references, occlusal view; (e) CBCT volume rendering with references, side view; (f) CBCT volume rendering with references, frontal view.

**Figure 2 fig2:**
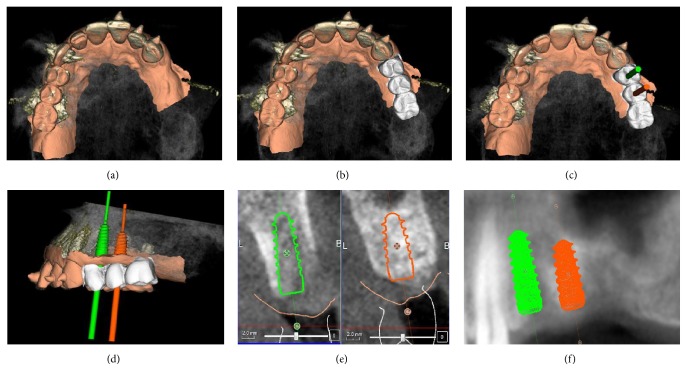
Surgical and prosthetic 3D planning: (a) overlapping of intraoral scan and CBCT; (b) overlapping of intraoral scan and CBCT with modelled provisional restorations; (c) overlapping of intraoral scan and CBCT with modelled provisional restoration and implant planning (occlusal view); (d) overlapping of intraoral scan and CBCT with modelled provisional restoration and implant planning (side view); (e) implant planning (cross sections); (f) implant planning (panorex).

**Figure 3 fig3:**
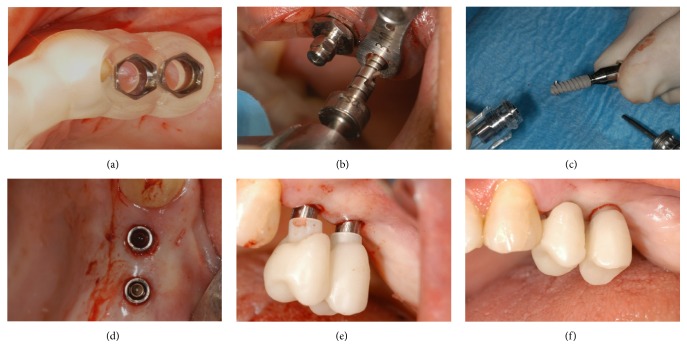
Surgery and immediate provisionalization: (a) the surgical guide in position; (b) preparation of the surgical sites; (c) implant placement; (d) all implants are placed with a flapless procedure; (e) the provisional PMMA restoration is placed on the temporary titanium abutments; (f) the provisional PMMA is screwed on the implants.

**Figure 4:**
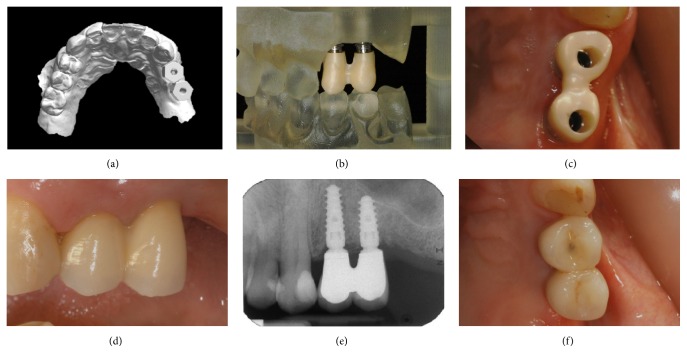
Three months after implant placement, the definitive intraoral impression is taken, and the definitive implant-supported restoration is fabricated and delivered to the patient: (a) digital impression with PEEK scanbodies; (b) the 3D-printed model with the zirconia framework; (c) the zirconia framework is seated on the definitive titanium abutments and checked for accuracy/precision (occlusal view); (d) delivery of the final zirconia-ceramic restoration, cemented on the final titanium abutments (side view); (e) periapical radiograph at the delivery of the final implant-supported restoration; (f) 6-month control of the final zirconia-ceramic restoration (occlusal view).

Fifteen patients (10 males and 5 females, aged between 26 and 70 years, average age 51.5 ± 12.0) requesting oral rehabilitation with fixed, implant-supported prostheses in the posterior region of the maxilla were selected and recruited to participate in this prospective clinical study. Following an image-capturing procedure using intraoral scanning and CBCT, the surgical planning was done with dedicated software. Surgical guides were then fabricated for placing the implants using 3D printing; temporary protheses for immediate loading were milled in PMMA. Each patient received two implants through a guided surgical procedure, with placement of the temporary prosthesis at the same surgical session and immediate loading. The surgical guides were easily positioned on the supporting teeth and were sufficiently stable. In all, 30 implants were placed using a flapless procedure. The implants were immediately loaded with fixed partial prostheses made of PMMA. These prostheses were easily adjusted on temporary titanium abutments, which were screwed in and remained* in situ* for a total period of three months. In the week following the intervention, the patients did not report any postsurgical discomfort or pain and were extremely pleased with both the aesthetic and functional aspects of the loaded restorations. At the end of the temporary period, the final optical impression was taken and frameworks were milled from zirconia, and 3D-printed resin models were fabricated. The frameworks were found to be sufficiently accurate at an intraoral test and were returned to the technician for ceramic coating and aesthetic finalisation. After 1 week, the zirconia-ceramic prostheses were delivered to the patient and cemented onto the permanent titanium abutments. At a follow-up check 6 months later, no patient reported having had any problems or biological or functional complications resulting from the implant-supported restorations. All the prostheses were functioning and patients were all extremely satisfied (Figures 1 [Fig fig2] [Fig fig3]–4).

## 4. Discussion

The ability to plan the insertion of dental implants virtually and subsequently place the fixtures in the exact position at the desired depth and angle using accurately milled or 3D-printed surgical guides has long been a clinical reality [2, 3, 5, 12]. Guided surgery has been a successful procedure for over 10 years, as evidenced by several clinical studies [12, 13] and systematic reviews [3, 14, 15]. Initially, the use of guided surgery techniques was limited to complex cases (patients who were fully edentulous, with manufacture of bone-supported or mucosa-supported templates); in fact, in order to obtain bone anatomy information, the patient had to be subjected to conventional computerised axial tomography (CT scanning), involving exposure to significant amounts of ionising radiation [3, 14, 15].

This is all changed now. The introduction of cone beam computed tomography (CBCT), which can capture 3D information on bone anatomy with low doses of radiation, has greatly expanded the potential applications of guided surgery [4, 16]. These applications now extend to teeth-supported surgical guides and therefore to cases where the planning requires placement of a lower number of fixtures. The introduction of intraoral scanners, powerful tools for obtaining dental and gingival information, is a further development in available techniques for capturing images for surgical planning [6–9]. In fact these machines enable all the dental and gingival information required to be obtained with a beam of light [7, 8] and with an accuracy, precision, and image resolution significantly higher than that obtained from CT (and even CBCT). The information obtained can be easily combined and superimposed on bone architecture information, thanks to open reverse-engineering software or proprietary software [11, 12]. It is therefore possible to create a virtual model of the patient, containing all the information required (bone, tooth, and gum) to carry out the intervention for implant placement using surgical guides supported on the teeth [12].

For this prospective study, 15 patients were selected and treated with insertion of 30 implant fixtures using guided surgical procedures. The 3D-printed surgical guides were sufficiently stable, and the interventions proceeded quickly and smoothly using a flapless technique. The potential to operate without lifting the flap is the major biological advantage to guided surgery, resulting in immediate benefit for the patient [17, 18]. Postoperative inconvenience and discomfort are substantially reduced, even eliminated entirely, using the flapless method [18]. The implants were placed without difficulty and immediately loaded with temporary PMMA prostheses obtained by milling; these prostheses were sent to the dentist before surgery and placement of the implants. The ability to load implants immediately is a further major advantage of the method used in this study. The adjustment of preformed shells and temporaries can be time-consuming in conventional procedures for immediate loading [19]; the time it takes to reline, adapt, refine, and polish the temporary restorations necessarily entails discomfort for the patient, who needs to go home and rest after the surgery [20, 21]. Modern implant and prosthetic planning techniques can greatly reduce operating times, as in this study, where the temporary PMMA restorations were placed easily with no need for relining and often requiring only minor adjustment. The temporaries remained* in situ* for a total period of 3 months and were subsequently replaced with permanent ceramic-zirconia prostheses.

The implants were manufactured using a fully digital process. A second round of intraoral imaging was carried out after positioning modern transfer devices (scanbodies) made of an opaque material [22–24] in the mouth. These devices allowed the exact location of the implants to be transmitted to the virtual plan, to enable computer-assisted design (CAD) of the prosthetic structures (frameworks) in zirconia. Subsequently the zirconia structures were milled [25] and then tried in the mouth. After the trial, the technician could then apply ceramic to the structures with the aid of a 3D-printed resin model. The devices were delivered and adjusted for aesthetic and functional requirements, to the complete satisfaction of the patients. At the 6-month check-up, no problems of any biological or prosthetic nature were reported and all restorations were functional and under load.

This study is subject to limitations. First, the number of treated patients (and consequently the number of implants placed) was rather restricted; also, the patients were only checked 6 months after placing the permanent restorations. Further studies are certainly required to validate this technique for planning implants. Last but not least, the use of this technique for guided surgery is limited by the size of the mouth opening. It was not possible to apply the technique to all patients since the surgical instrumentation is bulky, and not all patients have a large enough opening to allow implants to be placed, especially in the molar area, and for this reason they were excluded from the study. New methods will no doubt be developed that are not restricted by problems of space, which will therefore enable the application of guided surgery techniques to be extended to all patients. Finally, the stability of 3D-printed surgical guides can still be a problem. Although their stability was satisfactory in this analysis, at least ideally, the surgical teeth-supported guides should rest on the teeth at certain points; the greater the area of support, in fact, the more complex it can be to obtain the perfect fit (e.g., on the occlusal surface). The elimination of undercuts is of great importance, and the size of the guide should be as reduced as possible, to avoid problems caused by contraction of the material over time, which can cause problematic misfits.

Nevertheless, this study has shown that it is now possible to plan and implement short-span prostheses supported by implants with a fully digital process, using surgical planning. This allows optimum placing of implants, reducing the risks linked with surgical intervention and the time it takes. Postoperative discomfort for the patient is greatly reduced by using the flapless method. The full digital process also allows significant time savings for the patient and the practitioner, resulting in reduced [26, 27] costs.

## 5. Conclusions

In this study we have presented a fully digital method for the guided placing of implants in the posterior region of the maxilla of 15 patients and the subsequent fabrication of implant-supported fixed prostheses. In all, 30 implants were placed using a flapless procedure. The surgical guides were easily positioned on the supporting teeth and were sufficiently stable. The implants were immediately loaded with fixed partial prostheses made of PMMA. These prostheses were easily adjusted on temporary titanium abutments, which were screwed in and remained* in situ* for a total period of three months. At the end of the temporary period, a final optical impression was taken, frameworks were milled from zirconia, and 3D-printed resin models were fabricated. The frameworks were then coated with ceramic and delivered to the patients. At a follow-up check 6 months later, no patient reported having had any problems or biological or functional complications resulting from the implant-supported restorations. All the prostheses were functioning and patients were all extremely satisfied. The study is subject to limitations (small number of patients and implants, short follow-up period), and further studies will be needed to validate the method presented here.
